# 

**Published:** 1898-06-18

**Authors:** 


					THE HOSPITAL. "The standard of highest purity."?Lancet, June 18th, 1898.
CADBURY'S s?^ UT ^ CADBURY'S 1
COGOA llrll /M^ COCOA
^ ABSOLUTELY PURE. ? f*1- i NO DRUGS OR ALKALIES USED.
WHICH HOSPl.TAL
No. 612. Vol. XXIV. GXSjfp^] Sattodat,
June 18th, 1898.
[8ub3lm. J:?ICEF TWOPENCE.

				

